# Transmission of chimeric HIV by mating in conventional mice: prevention by pre-exposure antiretroviral therapy and reduced susceptibility during estrus

**DOI:** 10.1242/dmm.012617

**Published:** 2013-07-25

**Authors:** Eran Hadas, Wei Chao, Hongxia He, Manisha Saini, Eleen Daley, Mohammed Saifuddin, Galina Bentsman, Eric Ganz, David J. Volsky, Mary Jane Potash

**Affiliations:** 1 Molecular Virology Division, St Luke’s-Roosevelt Hospital Center, Columbia University Medical Center, New York, NY 10019, USA; 2 CONRAD, Eastern Virginia Medical School, 1911 Fort Myer Drive, Suite 900, Arlington, VA 22209, USA; 3 Obstetrics and Gynecology, St Luke’s-Roosevelt Hospital Center, Columbia University Medical Center, New York, NY 10019, USA

## Abstract

Heterosexual transmission accounts for the majority of new human immunodeficiency virus (HIV) cases worldwide. The current approach to investigate HIV heterosexual transmission in animals involves application of virus stock to the vaginal surface, a method that does not reproduce the physiological conditions of vaginal intercourse that influence the rate of transmission. We have previously described efficient infection of conventional mice using EcoHIV/NL4-3 and EcoHIV/NDK, chimeric HIV molecular clones constructed to express all HIV structural and regulatory genes except envelope, which is replaced by a rodent-tropic envelope gene. Here we investigated whether EcoHIV/NDK-infected male mice transmit virus to females during coitus, and the sensitivity of this transmission to HIV pre-exposure prophylaxis and the estrus state. Our general approach was to allow mating between EcoHIV/NDK-infected male mice and uninfected females for 1–7 nights. At 1–6 weeks after mating, mice were euthanized and virus burdens were measured by quantitative PCR (qPCR) amplification of HIV RNA or DNA in peritoneal macrophages, inguinal lymph node cells, spleen cells or vas deferens, or by ELISA for antibodies to HIV Gag. We found that 70–100% of female mice mated to EcoHIV/NDK-infected males acquired infection. Pericoital treatment of females with either 2′,3′-dideoxcytidine (ddC) or tenofovir largely prevented their EcoHIV/NDK infection by mating (*P*<0.05 and *P*<0.003, respectively). In males, T cells were dispensable for virus transmission. The rate of EcoHIV/NDK sexual transmission to females in estrus declined sharply (*P*=0.003) but their infection by injection was unaffected, indicating that the local environment in the female reproductive tract influences susceptibility to HIV. We conclude that this system of EcoHIV/NDK transmission during mouse mating reproduces key features of heterosexual transmission of HIV in humans and can be used to investigate its biology and control.

## INTRODUCTION

Since the recognition of acquired immune deficiency syndrome (AIDS), animal models have been developed to study various aspects of human immunodeficiency virus (HIV) infection, pathogenesis and control. The most common route of HIV transmission, sexual transmission (UNAIDS, 2012), has been investigated by simian immunodeficiency virus (SIV) infection of macaques and HIV infection of humanized mice. For infection, virus stock is applied to the female reproductive tract (FRT) or rectum, often after a course of progestin (Cranage et al., 2008; Denton et al., 2008; Denton et al., 2011; Miller et al., 2005; Sun et al., 2007). These animal models have shown the ability of some antiretrovirals in topical application or injection to prevent or reduce vaginal or rectal virus infection (Cranage et al., 2008; Denton et al., 2008; Denton et al., 2011; Sun et al., 2007). Physiological factors that can influence viral sexual transmission that are absent from these formats are semen as the inoculum, female hormonal cycle changes in susceptibility to infection, and the responses of the FRT to copulation. Semen from HIV-infected men contains cell-free virus and infected cells, and each can mediate infection (Quayle et al., 1997). Cytokines in semen can activate cells of the FRT and the levels of cytokines are higher during acute infection, when HIV sexual transmission is most efficient (Kafka et al., 2012; Lisco et al., 2012; Wawer et al., 2005). Seminal fluid contains enhancers and inhibitors of HIV infection (Münch et al., 2007; Sabatté et al., 2011) that can drive cytokine responses in the FRT that mediate chemotaxis of HIV target cells, possibly amplifying infection (Robertson, 2005). It could be valuable to reproduce these aspects of exposure to HIV that are specific to copulation in model systems.

Our animal model of HIV infection employs EcoHIV, chimeric HIV clones in which the gp120 coding region is replaced by the coding region of the ecotropic murine leukemia virus (MLV) envelope, switching the viral tropism from human to rodent (Potash et al., 2005); all other genes and functions in EcoHIV are derived from HIV. Two viruses have been constructed on Subtype B and Subtype D HIV backbones, EcoHIV/NL4-3 and EcoHIV/NDK, respectively. After inoculation into conventional mice, the viruses infect lymphocytes and macrophages; antiretrovirals, HIV DNA vaccination and anti-EcoHIV CD8+ T cells each can control this virus replication in mice (Hadas et al., 2007; Kelschenbach et al., 2012; Roshorm et al., 2009; Saini et al., 2007).

**RESOURCE IMPACT**

**Background**

Human immunodeficiency virus (HIV), which causes acquired immunodeficiency syndrome (AIDS), is primarily transmitted via heterosexual intercourse. Constituents of seminal fluid drive cytokine responses in the female reproductive tract that can amplify HIV infection. Moreover, levels of immunity in the female reproductive tract vary over the course of the menstrual cycle and susceptibility to virus infection is influenced by sex hormones, demonstrating the importance of the physiological environment. Although HIV infection and pathogenesis have been modeled fairly extensively in animal models, the currently available models do not allow the dynamic features of viral sexual transmission to be examined. To address this limitation, the present study describes a system for HIV transmission during mating.

**Results**

To generate a system for viral sexual transmission, chimeric HIV molecular clones (EcoHIV) were constructed and used to infect rodents. The authors demonstrate that EcoHIV-infected male mice efficiently transmit the virus by mating to uninfected females. They show that infection of females can be detected by the presence of antibodies against HIV, or of HIV DNA or RNA in lymphocytes and macrophages. In addition, exposure of females to antiretroviral drugs around the time of mating prevents their infection. Finally, the authors report that female mice in estrus lose susceptibility to EcoHIV sexual transmission yet retain susceptibility to infection via injection.

**Implications and future directions**

The findings reported here indicate that HIV sexual transmission can be modeled by mating conventional uninfected mice to mice infected with EcoHIV. This novel approach creates the possibility of studying the requirements for sexual transmission of HIV using the extensive repertoire of available genetically engineered and mutant mice while integrating the influences of the male and female mammalian reproductive tissues on infection. Highlighting this potential, the authors demonstrate that the rate of viral transmission declines sharply during estrus, indicating that the local environment in the female reproductive tract influences viral infectivity. Most importantly, the work provides a simple, small animal platform to investigate interventions to prevent the most frequent route of HIV transmission.

Given the ability to investigate HIV replication in conventional mice and the pivotal observation that male to female transmission of retroviruses in mice is almost exclusively through sexual transmission (Portis et al., 1987), this study was conducted to investigate EcoHIV/NDK sexual transmission and control in mice. We report that female mice acquired EcoHIV/NDK infection by mating to infected males. This transmission can largely be prevented by pericoital treatment of females with antiretroviral drugs, and transmission efficiency declines during estrus.

## RESULTS

**Fig. 1.**
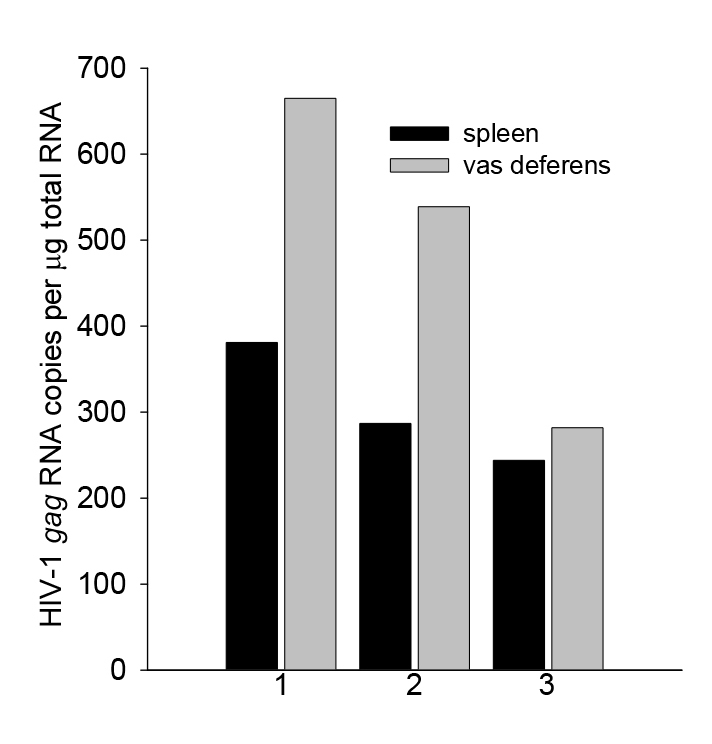
**EcoHIV/NDK burden in spleen and vas deferens of infected mice.** Male mice were inoculated with EcoHIV/NDK and euthanized 2 weeks later. Spleen and vas deferens were collected, and RNA was isolated and subjected to quantitative PCR (qPCR) amplifying HIV NDK *gag*. Bars represent average values from the three individual mice.

HIV is present in human semen and the titer in semen correlates with the efficiency of sexual transmission (Pilcher et al., 2007). To investigate EcoHIV/NDK expression in male reproductive tissue compared with lymphoid tissue, we evaluated HIV *gag* RNA burden in the vas deferens and spleen 2 weeks after mouse infection by injection (Fig. 1). HIV RNA was detected at similar levels in both organs; this observation and the previous finding that lymphocytes from EcoHIV/NL4-3-infected mice produce transmissible virus (Potash et al., 2005) indicate that it is likely that the vas deferens can provide infectious virus for sexual transmission.

**Fig. 2.**
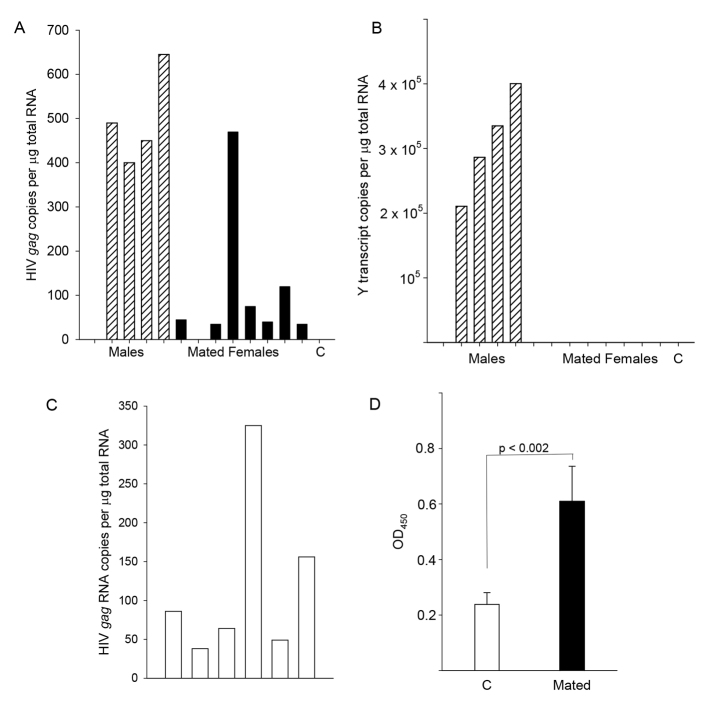
**EcoHIV/NDK-infected male mice transmit virus by mating.** (A,B) At 1 week after mating, PMs were collected from male and female mice, and RNA was isolated and subjected to qPCR amplifying HIV/NDK *gag* (A) or the Y chromosome transcript (B). Bars represent average, normalized values from individual mice. ‘C’, control; striped bars, males; black bars, mated females. (C,D) Infection, mating, and assay of EcoHIV/NDK RNA burden in female PMs 1 week after mating were repeated (C) and females were euthanized 6 weeks after mating for collection of plasma and assay of anti-HIV Gag antibodies by ELISA (D). (D) Data presented show mean ± standard error of OD_450_, detecting Gag binding IgG in plasma from four control females (‘C’) and six female mice after breeding (Mated).

EcoHIV/NDK-infected male mice were caged with uninfected female mice for 7 nights prior to tissue collection. C57BL/6 mice were used because they mated more efficiently than other strains in our colony. All mice were euthanized, and HIV *gag* transcripts and a ubiquitous Y chromosome transcript, to detect male tissue, were measured in peritoneal macrophages (PMs) (Fig. 2). Seven of eight females acquired EcoHIV infection by mating and the unmated control remained negative (Fig. 2A). Female cell extracts did not contain detectable male RNA, demonstrating that the virus that was detected did not reside in contaminating male cells (Fig. 2B).

Because mice infected by injection of EcoHIV/NL4-3 seroconvert (Potash et al., 2005), detection of anti-HIV antibodies in female mice after sexual transmission can provide additional evidence of infection and viral protein production. Female mice were caged for 1 night with EcoHIV/NDK-infected males and, after 1 week, PMs were collected to measure virus burden: HIV *gag* RNA was detected in all females (Fig. 2C). At 6 weeks after mating, plasma was collected from females and anti-HIV/NDK Gag antibodies were measured by ELISA; plasma from four uninfected mice was also tested (Fig. 2D). The anti-Gag titers were significantly greater (*P*<0.002) in infected, mated female mice. Thus, female mice can be infected by mating through limited exposure to infected males; they produce EcoHIV/NDK proteins and mount antiviral responses.

**Fig. 3.**
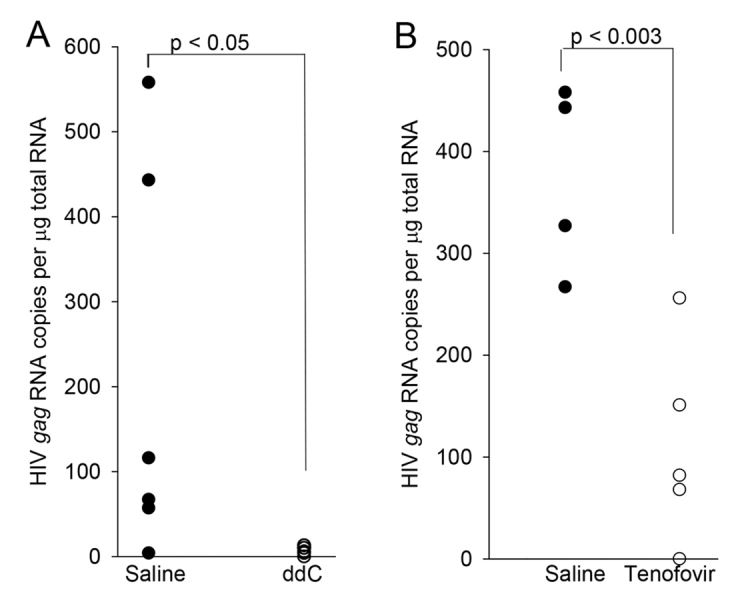
**Sexual transmission of EcoHIV/NDK can be blocked by antiretroviral administration to female mice.** (A) Female mice were injected with vehicle (black circles) or ddC (white circles; A) or vehicle or tenofovir (white circles; B) and exposed to EcoHIV/NDK-infected male mice as indicated in the text. Virus burden in female PMs was determined as described in Fig. 2. Each point represents average normalized values from a single mouse.

Pre-exposure prophylaxis (PrEP) in human beings can prevent HIV sexual transmission (Celum and Baeten, 2012; Grant et al., 2010) and antiretroviral drugs can prevent EcoHIV/NDK infection from injection in mice (Hadas et al., 2007). PrEP with 2′,3′-dideoxcytidine (ddC) or tenofovir was tested for its efficacy to prevent sexual transmission of EcoHIV/NDK. Female mice were treated every 12 hours by intraperitoneal (IP) injection of ddC or vehicle, starting 36 hours prior to caging with EcoHIV/NDK-infected male mice, similar to studies in humanized mice (Denton et al., 2008). Females were exposed to males for 5 nights and were euthanized 1 week after beginning mating. HIV *gag* burden was measured in PMs (Fig. 3A). EcoHIV was transmitted to five out of six vehicle-treated females and to none of five females treated with ddC, indicating that an antiretroviral can prevent EcoHIV transmission by mating (*P*<0.05). We then limited antiretroviral treatment to two injections and also limited female exposure to infected males to 1 night. Females were treated with tenofovir or vehicle and 12 hours later were housed with EcoHIV/NDK-infected males overnight. The next morning females received another injection and were caged separately from males. At 1 week after exposure to males, females were euthanized and virus burden was measured in PMs (Fig. 3B). Pericoital tenofovir treatment largely prevented EcoHIV infection with statistical significance of *P*<0.003, analogous to its effect in the prevention of HIV sexual transmission in some humans (Celum and Baeten, 2012; Grant et al., 2010).

**Fig. 4.**
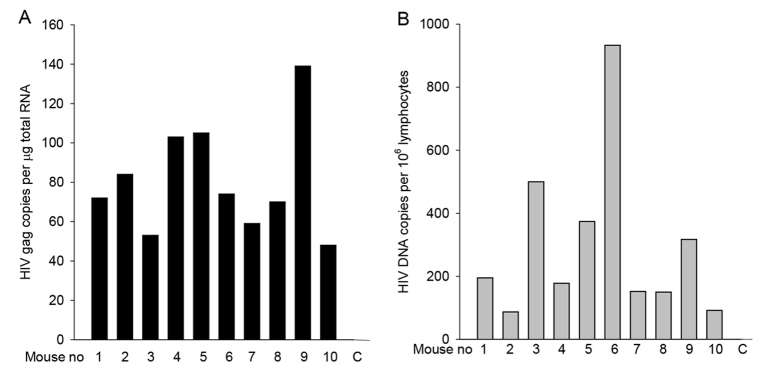
**T cells are dispensable for EcoHIV/NDK sexual transmission from infected male mice.** Nude male mice were infected with EcoHIV/NDK and caged with C57BL/6 females; females were euthanized, tissues collected, and virus RNA burden in PMs (A) and viral DNA burden in inguinal lymph node cells (B) determined as described in Fig. 2 and the text. ‘C’, control.

With the ability to study HIV sexual transmission through mating infected mice to uninfected mice, we investigated the requirement for T cells by the use of athymic nude (*Foxn1^nu^*) male mice, in which T cell development is impaired (Wortis et al., 1971). Nude mice are susceptible to EcoHIV/NDK infection by injection (E.H. and D.J.V., unpublished). EcoHIV/NDK-infected nude males were caged with C57BL/6 females during the nights for 1 week, then females were euthanized to collect PMs and inguinal lymph nodes. Viral RNA was measured in PMs (Fig. 4A) and viral DNA was measured in inguinal lymph nodes to detect an early product of infection (Fig. 4B). All ten mated females carried HIV DNA in lymphocytes and HIV RNA in PMs. We conclude that male mice do not require T cells for sexual transmission of EcoHIV/NDK.

**Fig. 5.**
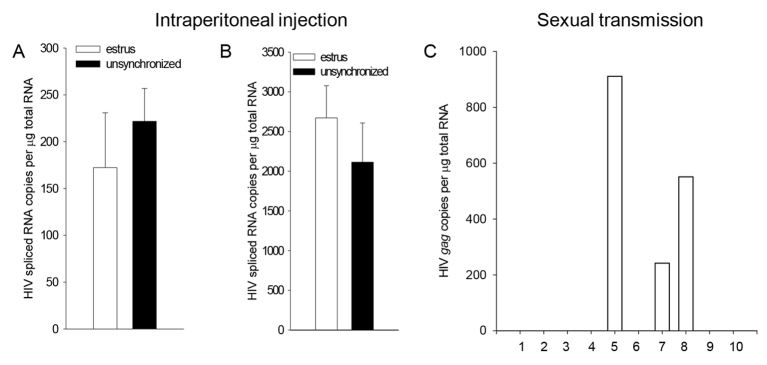
**The efficiency of sexual transmission of EcoHIV/NDK to female mice declines during estrus.** (A,B) Female mice were synchronized in estrus (white bar) or left unsynchronized (black bar) and infected with EcoHIV/NDK by IP injection. Virus burden was measured 1 week after infection by amplification of HIV *vif* transcripts in splenic lymphocytes (A) or PMs (B). Bars represent the mean ± standard error of values from six mice in estrus and four unsynchronized females. (C) Female mice in estrus were caged with EcoHIV-infected male mice, euthanized and virus burden in PMS determined as described in Fig. 2.

Vaginal susceptibility to HIV in women or to SIV in macaques varies as a result of menstrual cycle or sex hormone administration, with greater susceptibility associated with higher levels of natural or synthetic progesterone; conversely topical administration of estrogen prevents vaginal infection by SIV (Heffron et al., 2012; Smith et al., 2004; Vishwanathan et al., 2011). Female mice can be reliably synchronized in estrus by exposure to male urine (Marsden and Bronson, 1964), permitting evaluation of susceptibility to EcoHIV/NDK infection as it varies with natural hormonal cycling in the FRT. To investigate the effects of estrus on the transmission of EcoHIV/NDK, groups of female mice synchronized in estrus were either caged with EcoHIV/NDK-infected male mice or injected with EcoHIV/NDK stock. Some unsynchronized females were infected by injection for comparison. All mice were euthanized 1 week after virus exposure (Fig. 5). Females synchronized in estrus and unsynchronized females were equally susceptible to EcoHIV/NDK infection by injection (Fig. 5A,B). In sharp contrast, females in estrus were much less susceptible to EcoHIV/NDK sexual transmission than unsynchronized females, with only three of ten mated females infected (Fig. 5C; for comparison see Figs 2–4); however, females in estrus mated efficiently, with nine of ten becoming pregnant. These findings indicate that estrus reduces the susceptibility of female mice to EcoHIV/NDK sexual transmission locally, without systemic effects upon infection. They also provide clear evidence that sexual transmission and not casual contact is responsible for EcoHIV/NDK transmission from infected male to female mice.

## DISCUSSION

The results presented in this work demonstrate that EcoHIV/NDK-infected male mice transmit virus efficiently to female mice by mating. To our knowledge, this is the first description of HIV transmission by coitus in an animal model. The current approaches for experimental study of HIV sexual transmission involve application of concentrated SIV or HIV stocks to vaginal or rectal surfaces in macaques or humanized mice (Cranage et al., 2008; Denton et al., 2008; Denton et al., 2011; Miller et al., 2005; Sun et al., 2007). Both in humanized mice and the mouse mating model described here, virus introduced into the FRT causes systemic infection (Denton et al., 2008; Denton et al., 2011) (Figs 2–4); however, we believe that EcoHIV/NDK transmission by mating can reveal new insights into the physiological conditions in partners of either sex that control the rate and efficiency of HIV sexual transmission. Several points should be made about our findings:

First, HIV transmission by mating in mice was reproducible and highly efficient, with the majority of females showing evidence of infection (Figs 2–4). The efficiency of sexual transmission of EcoHIV/NDK is not surprising: 25 years ago investigators found that sexual transmission was the predominant mode of MLV transmission from infected male to female mice (Portis et al., 1987). Indeed, mating of infected to uninfected mice has been used previously to study transmission of LP-BM5 MLV as a model for HIV sexual transmission (Jones et al., 2012; Okada et al., 1998). Like HIV and murine retroviruses (Jones et al., 2012; Pilcher et al., 2007), EcoHIV/NDK is easily detected in the male reproductive tissue (Fig. 1). The observed HIV or MLV sexual transmission rates in mice were significantly higher than those reported for HIV in humans (Wawer et al., 2005). Although the factors contributing to the higher rate are still being defined, using males at or near the peak of infection and using females outside estrus are likely to play roles (Fig. 5). At the practical level, high rates of EcoHIV/NDK sexual transmission in mice are essential for statistically reliable evaluation of interventions to block this transmission (Fig. 3).

Second, EcoHIV transmission during mouse mating preserves the features of the male and female reproductive tracts, including host factors and cells in seminal fluid that accompany virions from males and those encountered by incoming virus in females. One aspect of mechanisms of HIV sexual transmission can be seen in our use of infected male athymic mice in mating (Fig. 4). These mice lack T cells (Wortis et al., 1971) and, given their ability to transmit virus, our data suggest that T cells are dispensable for transmission of EcoHIV/NDK from males. In this case, the abundant monocytes that are present in the murine male reproductive tract likely contribute virus for sexual transmission (Mullen et al., 2003). Semen contains fragments of prostatic acidic phosphatase that enhance HIV infection (Münch et al., 2007). Soon after HIV infection, semen carries cytokines and β-chemokines that can induce macrophage chemotaxis to the FRT (Kafka et al., 2012), including transforming growth factor β, which is found in semen of uninfected humans and mice (Sharkey et al., 2012a). In tissue culture, semen also induces inflammatory cytokines in human endometrial cells that in turn activate the HIV-1 long terminal repeat (LTR), providing a mechanism to locally amplify virus transmission (Kafka et al., 2012). Coitus itself affects the FRT, generating a ‘post-mating inflammatory cascade’ observed in women and female mice, with cytokine and chemokine induction and the migration of macrophages and dendritic cells (Robertson, 2005; Sharkey et al., 2012b). The influx of potential target cells into the FRT might contribute to the observed efficiency of EcoHIV infection. These dynamic features of copulation contributed by both the male and female partners are not reproduced by application of virus stock to the vaginal surface (Miller et al., 2005; Sun et al., 2007). Our goal in establishing the system of chimeric HIV transmission by mouse mating was to preserve these features of copulation for integration of the possible effects of the cells, tissues and responses that affect infection.

Also intrinsic to our model of HIV sexual transmission is the construction of EcoHIV itself; it preserves the entire HIV genome, except gp120. Thus, the functions of or interventions against any of the other HIV proteins or the LTR can be assessed in EcoHIV-infected mice, as exemplified in Fig. 3. This potential utility of this construction is indicated in the observation that SIV reverse transcriptase is relatively resistant to non-nucleoside reverse transcriptase inhibitors (NNRTI) and SIV constructs encoding HIV reverse transcriptase were required to test NNRTI in macaques (Witvrouw et al., 2004). In a similar fashion, EcoHIV constructs encode HIV Vif, which restricts the innate immune effector APOBEC3G to promote HIV infection (Sheehy et al., 2002). An interesting recent study demonstrated that mouse APOBEC3 reduces the sexual transmission of the murine retrovirus swarm LP-BM5, which has been used as a model of HIV (Jones et al., 2012). However, this LP-BM5 does not carry Vif and the antagonism of APOBEC3 and Vif that is present in HIV infection could not be examined.

EcoHIV/NDK sexual transmission was sensitive to antiretrovirals in PrEP (Fig. 3), echoing findings in humans. Oral tenofovir or tenofovir plus emtricitabine prevented HIV transmission between men and within discordant heterosexual couples in some but not all trials (Celum and Baeten, 2012; Grant et al., 2010). In other prevention strategies, vaccination with different HIV DNA constructs has been shown to induce protective immunity and prevent EcoHIV/NL4-3 infection by injection (Im et al., 2011; Roshorm et al., 2009; Saini et al., 2007). We believe that EcoHIV transmission by mouse mating can complement the existing preclinical screens of HIV prevention through antiretrovirals or vaccination to bring only the most promising approaches to clinical trial.

Finally, the rate of EcoHIV/NDK sexual transmission was greatly reduced in mice in estrus although their overall susceptibility to EcoHIV/NDK infection was not affected (Fig. 5), indicating the importance of the local hormonal environment in the FRT in controlling virus susceptibility. Vaginal transmission of other viruses is under similar control. Mice in estrus are also resistant to vaginal transmission of herpes simplex virus type 2 (Gallichan and Rosenthal, 1996). With peak estradiol levels occurring in mice at estrus and rapidly declining as progesterone levels rise after ovulation (Curtis et al., 1999), our findings are consistent with observations that estrogens tend to prevent and progesterone tends to promote HIV or SIV infection. Topical estrogen can largely prevent vaginal infection by SIV in macaques without affecting their susceptibility to infection by injection (Smith et al., 2004). Macaques are most susceptible to SIV vaginal infection at the peak of progesterone expression and are far less susceptible when progesterone is low (Vishwanathan et al., 2011). Women using injectable hormonal contraceptives containing synthetic progesterone are at enhanced risk for both acquisition and transmission of HIV during intercourse (Heffron et al., 2012). The changes in susceptibility to HIV or SIV vaginal infection have been attributed to changes in local immune effector mechanisms, epithelial wall thickness and pH, among others (Smith et al., 2004; Vishwanathan et al., 2011; Wira and Fahey, 2008). Given the ease of synchronizing mice in estrus, our system could be useful to investigate the contributors to cyclical differences in HIV susceptibility in the FRT. If such differences can be extended to women, they might provide a basis to avoid HIV sexual transmission during the period of greatest susceptibility.

The findings reported here indicate that HIV sexual transmission can be modeled by mating conventional uninfected mice to mice infected with EcoHIV/NDK, opening the extensive repertoire of genetically engineered and mutant mice to study the requirements for this transmission route, integrating the influences of male and female reproductive tissue upon infection, and providing a simple, small animal platform to investigate interventions to prevent the most frequent route of HIV transmission.

## MATERIALS AND METHODS

### Mice

Male and female C57BL/6 mice and *Foxn1^nu^* (nude) mice from 6–16 weeks of age were either purchased from the Jackson Laboratory (Bar Harbor, ME) or bred in our mouse colony. All use of mice was approved by the St Luke’s-Roosevelt Institutional Animal Care and Use Committee (IACUC). Most experiments reported here employed C57BL/6 mice; the use of nude mice is explicitly noted in the text.

### Viruses, infection, real-time PCR and ELISA

EcoHIV/NDK construction, preparation, mouse euthanasia and tissue collection were as described previously (Potash et al., 2005). Plasmid DNA encoding EcoHIV/NDK is available to qualified investigators upon completion of a Material Transfer Agreement. Mouse infection by IP injection of 10^6^ pg HIV p24, ddC or tenofovir administration by IP injection, and real-time PCR amplifying HIV *gag* DNA or *gag* or *vif* RNA, standardized by amplification of glyceraldehyde phosphate dehydrogenase, were as described previously (Hadas et al., 2007). An effective dose of ddC of 200 mg/kg body weight was found previously (Hadas et al., 2007) and tenofovir was administered at 20 mg/kg body weight based on studies of SIV vaginal transmission in macaques (García-Lerma et al., 2010). Every assay of virus burden included cell extracts from uninfected mice as negative controls. The murine Y chromosome transcript was amplified using TaqMan Gene Expression Assay Mm0047710_ml (ABI, Columbia, MD). ELISA to detect antibodies to HIV Gag was as described previously (Saini et al., 2007), except that HIV/NDK p55 was used in solid phase. HIV/NDK p55 was amplified by PCR, cloned into pET-28, expressed in *Escherichia coli* and purified by nickel-NTA affinity chromatography. HIV burden in groups of mice were compared using Student’s *t*-test.

### Mating

Female mice were caged in groups of five unless otherwise indicated. At 1 week after EcoHIV/NDK infection, individual male mice were housed overnight with two uninfected female mice. Males were removed the next morning and were returned to cages for 1 or more nights as indicated in the text. Synchronization of females in estrus was achieved through the Whitten effect (Marsden and Bronson, 1964; Whitten, 1956): females were housed in pairs in wood shavings from male cages for 1 week prior to housing with infected males. Pregnancy was determined upon necroscopy.

## References

[b1-0061292] Celum C. Baeten J. M. (2012). Tenofovir-based pre-exposure prophylaxis for HIV prevention: evolving evidence. Curr. Opin. Infect. Dis. 25, 51–57 22156901 10.1097/QCO.0b013e32834ef5ef PMC3266126

[b2-0061292] Cranage M. Sharpe S. Herrera C. Cope A. Dennis M. Berry N. C. H. Ham C. Heeney J. Rezk N. Kashuba A. (2008). Prevention of SIV rectal transmission and priming of T cell responses in macaques after local pre-exposure application of tenofovir gel. PLoS Med. 5, e157, discussion e157. 18684007 10.1371/journal.pmed.0050157 PMC2494562

[b3-0061292] Curtis S. W. Clark J. Myers P. Korach K. S. (1999). Disruption of estrogen signaling does not prevent progesterone action in the estrogen receptor alpha knockout mouse uterus. Proc. Natl. Acad. Sci. USA 96, 3646–3651 10097091 10.1073/pnas.96.7.3646 PMC22348

[b4-0061292] Denton P. W. Estes J. D. Sun Z. Othieno F. A. Wei B. L. Wege A. K. Powell D. A. Payne D. Haase A. T. Garcia J. V. (2008). Antiretroviral pre-exposure prophylaxis prevents vaginal transmission of HIV-1 in humanized BLT mice. PLoS Med. 5, e16 18198941 10.1371/journal.pmed.0050016 PMC2194746

[b5-0061292] Denton P. W. Othieno F. Martinez-Torres F. Zou W. Krisko J. F. Fleming E. Zein S. Powell D. A. Wahl A. Kwak Y. T. (2011). One percent tenofovir applied topically to humanized BLT mice and used according to the CAPRISA 004 experimental design demonstrates partial protection from vaginal HIV infection, validating the BLT model for evaluation of new microbicide candidates. J. Virol. 85, 7582–7593 21593172 10.1128/JVI.00537-11 PMC3147928

[b6-0061292] Gallichan W. S. Rosenthal K. L. (1996). Effects of the estrous cycle on local humoral immune responses and protection of intranasally immunized female mice against herpes simplex virus type 2 infection in the genital tract. Virology 224, 487–497 8874509 10.1006/viro.1996.0555

[b7-0061292] García-Lerma J. G. Paxton L. Kilmarx P. H. Heneine W. (2010). Oral pre-exposure prophylaxis for HIV prevention. Trends Pharmacol. Sci. 31, 74–81 19963288 10.1016/j.tips.2009.10.009

[b8-0061292] Grant R. M. Lama J. R. Anderson P. L. McMahan V. Liu A. Y. Vargas L. Goicochea P. Casapía M. Guanira-Carranza J. V. Ramirez-Cardich M. E. iPrEx Study Team (2010). Preexposure chemoprophylaxis for HIV prevention in men who have sex with men. N. Engl. J. Med. 363, 2587–2599 21091279 10.1056/NEJMoa1011205 PMC3079639

[b9-0061292] Hadas E. Borjabad A. Chao W. Saini M. Ichiyama K. Potash M. J. Volsky D. J. (2007). Testing antiretroviral drug efficacy in conventional mice infected with chimeric HIV-1. AIDS 21, 905–909 17457083 10.1097/QAD.0b013e3281574549

[b10-0061292] Heffron R. Donnell D. Rees H. Celum C. Mugo N. Were E. de Bruyn G. Nakku-Joloba E. Ngure K. Kiarie J. Partners in Prevention HSV/HIV Transmission Study Team (2012). Use of hormonal contraceptives and risk of HIV-1 transmission: a prospective cohort study. Lancet Infect. Dis. 12, 19–26 21975269 10.1016/S1473-3099(11)70247-X PMC3266951

[b11-0061292] Im E.-J. Hong J. P. Roshorm Y. Bridgeman A. Létourneau S. Liljeström P. Potash M. J. Volsky D. J. McMichael A. J. Hanke T. (2011). Protective efficacy of serially up-ranked subdominant CD8+ T cell epitopes against virus challenges. PLoS Pathog. 7, e1002041 21625575 10.1371/journal.ppat.1002041 PMC3098219

[b12-0061292] Jones P. H. Mehta H. V. Okeoma C. M. (2012). A novel role for APOBEC3: susceptibility to sexual transmission of murine acquired immunodeficiency virus (mAIDS) is aggravated in APOBEC3 deficient mice. Retrovirology 9, 50 22691411 10.1186/1742-4690-9-50 PMC3418182

[b13-0061292] Kafka J. K. Sheth P. M. Nazli A. Osborne B. J. Kovacs C. Kaul R. Kaushic C. (2012). Endometrial epithelial cell response to semen from HIV-infected men during different stages of infection is distinct and can drive HIV-1-long terminal repeat. AIDS 26, 27–36 22095191 10.1097/QAD.0b013e32834e57b2

[b14-0061292] Kelschenbach J. L. Saini M. Hadas E. Gu C.-J. Chao W. Bentsman G. Hong J. P. Hanke T. Sharer L. R. Potash M. J. (2012). Mice chronically infected with chimeric HIV resist peripheral and brain superinfection: a model of protective immunity to HIV. J. Neuroimmune Pharmacol. 7, 380–387 21987348 10.1007/s11481-011-9316-1 PMC3487595

[b15-0061292] Lisco A. Munawwar A. Introini A. Vanpouille C. Saba E. Feng X. Grivel J. C. Singh S. Margolis L. (2012). Semen of HIV-1-infected individuals: local shedding of herpesviruses and reprogrammed cytokine network. J. Infect. Dis. 205, 97–105 22107749 10.1093/infdis/jir700 PMC3242745

[b16-0061292] Marsden H. M. Bronson F. H. (1964). Estrous synchrony in mice: alteration by exposure to male urine. Science 144, 1469 14171544 10.1126/science.144.3625.1469

[b17-0061292] Miller C. J. Li Q. Abel K. Kim E.-Y. Ma Z.-M. Wietgrefe S. La Franco-Scheuch L. Compton L. Duan L. Shore M. D. (2005). Propagation and dissemination of infection after vaginal transmission of simian immunodeficiency virus. J. Virol. 79, 9217–9227 15994816 10.1128/JVI.79.14.9217-9227.2005 PMC1168785

[b18-0061292] Mullen T. E. Jr Kiessling R. L. Kiessling A. A. (2003). Tissue-specific populations of leukocytes in semen-producing organs of the normal, hemicastrated, and vasectomized mouse. AIDS Res. Hum. Retroviruses 19, 235–243 12689416 10.1089/088922203763315740

[b19-0061292] Münch J. Rücker E. Ständker L. Adermann K. Goffinet C. Schindler M. Wildum S. Chinnadurai R. Rajan D. Specht A. (2007). Semen-derived amyloid fibrils drastically enhance HIV infection. Cell 131, 1059–1071 18083097 10.1016/j.cell.2007.10.014

[b20-0061292] Okada Y. Abe E. Komuro K. Mizuochi T. (1998). Heterosexual transmission of a murine AIDS virus. J. Virol. 72, 2541–2543 9499121 10.1128/jvi.72.3.2541-2543.1998 PMC109560

[b21-0061292] Pilcher C. D. Joaki G. Hoffman I. F. Martinson F. E. A. Mapanje C. Stewart P. W. Powers K. A. Galvin S. Chilongozi D. Gama S. (2007). Amplified transmission of HIV-1: comparison of HIV-1 concentrations in semen and blood during acute and chronic infection. AIDS 21, 1723–1730 17690570 10.1097/QAD.0b013e3281532c82 PMC2673564

[b22-0061292] Portis J. L. McAtee F. J. Hayes S. F. (1987). Horizontal transmission of murine retroviruses. J. Virol. 61, 1037–1044 3029398 10.1128/jvi.61.4.1037-1044.1987 PMC254060

[b23-0061292] Potash M. J. Chao W. Bentsman G. Paris N. Saini M. Nitkiewicz J. Belem P. Sharer L. Brooks A. I. Volsky D. J. (2005). A mouse model for study of systemic HIV-1 infection, antiviral immune responses, and neuroinvasiveness. Proc. Natl. Acad. Sci. USA 102, 3760–3765 15728729 10.1073/pnas.0500649102 PMC553332

[b24-0061292] Quayle A. J. Xu C. Mayer K. H. Anderson D. J. (1997). T lymphocytes and macrophages, but not motile spermatozoa, are a significant source of human immunodeficiency virus in semen. J. Infect. Dis. 176, 960–968 9333154 10.1086/516541

[b25-0061292] Robertson S. A. (2005). Seminal plasma and male factor signalling in the female reproductive tract. Cell Tissue Res. 322, 43–52 15909166 10.1007/s00441-005-1127-3

[b26-0061292] Roshorm Y. Hong J. P. Kobayashi N. McMichael A. J. Volsky D. J. Potash M. J. Takiguchi M. Hanke T. (2009). Novel HIV-1 clade B candidate vaccines designed for HLA-B*5101(+) patients protected mice against chimaeric ecotropic HIV-1 challenge. Eur. J. Immunol. 39, 1831–1840 19585509 10.1002/eji.200939309

[b27-0061292] Sabatté J. Remes Lenicov F. Cabrini M. Rodriguez Rodrigues C. Ostrowski M. Ceballos A. Amigorena S. Geffner J. (2011). The role of semen in sexual transmission of HIV: beyond a carrier for virus particles. Microbes Infect. 13, 977–982 21767659 10.1016/j.micinf.2011.06.005

[b28-0061292] Saini M. Hadas E. Volsky D. J. Potash M. J. (2007). Vaccine-induced protection from infection of mice by chimeric human immunodeficiency virus type 1, EcoHIV/NL4-3. Vaccine 25, 8660–8663 18023943 10.1016/j.vaccine.2007.10.019 PMC2219693

[b29-0061292] Sharkey D. J. Macpherson A. M. Tremellen K. P. Mottershead D. G. Gilchrist R. B. Robertson S. A. (2012a). TGF-β mediates proinflammatory seminal fluid signaling in human cervical epithelial cells. J. Immunol. 189, 1024–1035 22706080 10.4049/jimmunol.1200005

[b30-0061292] Sharkey D. J. Tremellen K. P. Jasper M. J. Gemzell-Danielsson K. Robertson S. A. (2012b). Seminal fluid induces leukocyte recruitment and cytokine and chemokine mRNA expression in the human cervix after coitus. J. Immunol. 188, 2445–2454 22271649 10.4049/jimmunol.1102736

[b31-0061292] Sheehy A. M. Gaddis N. C. Choi J. D. Malim M. H. (2002). Isolation of a human gene that inhibits HIV-1 infection and is suppressed by the viral Vif protein. Nature 418, 646–650 12167863 10.1038/nature00939

[b32-0061292] Smith S. M. Mefford M. Sodora D. Klase Z. Singh M. Alexander N. Hess D. Marx P. A. (2004). Topical estrogen protects against SIV vaginal transmission without evidence of systemic effect. AIDS 18, 1637–1643 15280774 10.1097/01.aids.0000131393.76221.cc

[b33-0061292] Sun Z. Denton P. W. Estes J. D. Othieno F. A. Wei B. L. Wege A. K. Melkus M. W. Padgett-Thomas A. Zupancic M. Haase A. T. (2007). Intrarectal transmission, systemic infection, and CD4^+^T cell depletion in humanized mice infected with HIV-1. J. Exp. Med. 204, 705–714 17389241 10.1084/jem.20062411 PMC2118553

[b34-0061292] UNAIDS (2012). Report on the Global AIDS Epidemic, pp. 34 Geneva: UNAIDS

[b35-0061292] Vishwanathan S. A. Guenthner P. C. Lin C. Y. Dobard C. Sharma S. Adams D. R. Otten R. A. Heneine W. Hendry R. M. McNicholl J. M. (2011). High susceptibility to repeated, low-dose, vaginal SHIV exposure late in the luteal phase of the menstrual cycle of pigtail macaques. J. Acquir. Immune Defic. Syndr. 57, 261–264 21546848 10.1097/QAI.0b013e318220ebd3

[b36-0061292] Wawer M. J. Gray R. H. Sewankambo N. K. Serwadda D. Li X. Laeyendecker O. Kiwanuka N. Kigozi G. Kiddugavu M. Lutalo T. (2005). Rates of HIV-1 transmission per coital act, by stage of HIV-1 infection, in Rakai, Uganda. J. Infect. Dis. 191, 1403–1409 15809897 10.1086/429411

[b37-0061292] Whitten W. K. (1956). Modification of the oestrous cycle of the mouse by external stimuli associated with the male. J. Endocrinol. 13, 399–404 13345955 10.1677/joe.0.0130399

[b38-0061292] Wira C. R. Fahey J. V. (2008). A new strategy to understand how HIV infects women: identification of a window of vulnerability during the menstrual cycle. AIDS 22, 1909–1917 18784454 10.1097/QAD.0b013e3283060ea4 PMC2647143

[b39-0061292] Witvrouw M. Pannecouque C. Switzer W. M. Folks T. M. De Clercq E. Heneine W. (2004). Susceptibility of HIV-2, SIV and SHIV to various anti-HIV-1 compounds: implications for treatment and postexposure prophylaxis. Antivir. Ther. 9, 57–65 15040537

[b40-0061292] Wortis H. H. Nehlsen S. Owen J. J. (1971). Abnormal development of the thymus in “nude” mice. J. Exp. Med. 134, 681–692 15776569 10.1084/jem.134.3.681 PMC2139084

